# Is there any association between early trimester Triglyceride–glucose index and incidence of hypertensive disorder of pregnancy and adverse pregnancy outcomes?

**DOI:** 10.3389/fendo.2023.1093991

**Published:** 2023-03-06

**Authors:** Yali Pan, Su Zou, Yingjia Xu, Ruomin Di, Huafen Gu, Zhangsheng Wang, Xiang Wei, Chenxi Yang, Gaofeng Zhang

**Affiliations:** Department of Cardiology, Shanghai Fifth People’s Hospital, Fudan University, Shanghai, China

**Keywords:** triglyceride glucose index, hypertensive disorder of pregnancy, low birth weight, fetal distress, Insulin resisitance

## Abstract

**Background:**

Insulin resistance (IR) is a normal feature of pregnancy and plays a crucial role in the pathophysiology of hypertensive disorder of pregnancy (HDP). The triglyceride-glucose index (TyG index) has been shown as a simple and reliable alternative IR marker. This work aimed to investigate the association between the TyG index and the incidence of HDP and adverse pregnancy outcomes.

**Methods:**

From January 2016 to December 2018, 289 women with HDP and 861 women without HDP were recruited at Shanghai Fifth People’s Hospital, Fudan University to determine the relationship between the TyG index and the incidence of HDP and adverse pregnancy outcomes.

**Results:**

In the case-control study, the incidence of HDP was found to be significantly associated with the TyG index. Moreover, logistic regression indicated that the TyG index is an independent risk factor for HDP development and incidence of low birth weight (LBW) and fetal distress. In the cohort study, the results showed that the TyG index increased, there was a stepwise increase in HDP incidence, SBP, and DBP levels one week before delivery as well as in LBW and fetal distress incidence. The early trimester TyG index was positively associated with pre-pregnancy BMI, systolic blood pressure (SBP), and diastolic blood pressure (DBP) one week before delivery. Spline regression showed that there was a significant linear association between HDP incidence and early trimester TyG index when it was >8.5.

**Conclusions:**

This work suggested that the early trimester TyG index was closely associated with the development of HDP and adverse pregnancy outcomes.

## Background

Hypertensive disorder of pregnancy (HDP) is defined as a group of diseases coexisting with pregnancy and hypertension, including gestational hypertension, preeclampsia, eclampsia, chronic hypertension with preeclampsia, and chronic hypertension with pregnancy (1). Except for chronic hypertension, HDP occurred mostly after 20 weeks of gestation and returned to normal within 12 weeks after delivery. Over the past few decades, HDP affect 5%–10% of pregnancies worldwide (2). In China, HDP prevalence has been reported to be 5.55–5.57% in 2011 (3), and with the improvement of living standards and support for multiple births in recent years, the prevalence of HDP has increased to 7.2% in 2021 (4). As a group of common pregnancy complications, HDP can lead to many adverse pregnancy outcomes including preterm delivery, placental abruption, cesarean section, postpartum hemorrhage, and a higher incidence of low birth weight (LBW) and fetal distress (3).

Insulin resistance (IR) is a normal feature of pregnancy and becomes more severe as the pregnancy progresses. Previous studies have shown that IR plays a crucial role in the pathophysiology of HDP (5–9). The triglyceride-glucose index (TyG index), based on fasting glucose and triglycerides, has been shown as a simple and reliable surrogate measure to reflect IR compared with the euglycemic-hyperinsulinemic clamp (10–12), which is the ‘gold standard’ for evaluating IR. Also, compared with the Homeostatic Model Assessment of Insulin Resistance (HOMA-IR), the TyG index is more accessible in clinical practice. Moreover, recent studies have demonstrated that the TyG index presented a better performance than the HOMA-IR index in identifying patients with IR and was more strongly associated with arterial stiffness in patients with type 2 diabetes mellitus (T2DM) in comparison with the HOMA-IR index (13, 14). In addition, Zheng et al. reported that the TyG index is an independent risk factor for hypertension and has a dose-dependent relationship, which can independently predict hypertension events (15). To the best of our knowledge, the relationship between the TyG index and the incidence of HDP remains unclear. The purpose of this study was to investigate the association between the TyG index and the incidence of HDP and adverse pregnancy outcomes.

## Methods

### Study population

**Figure 1:**
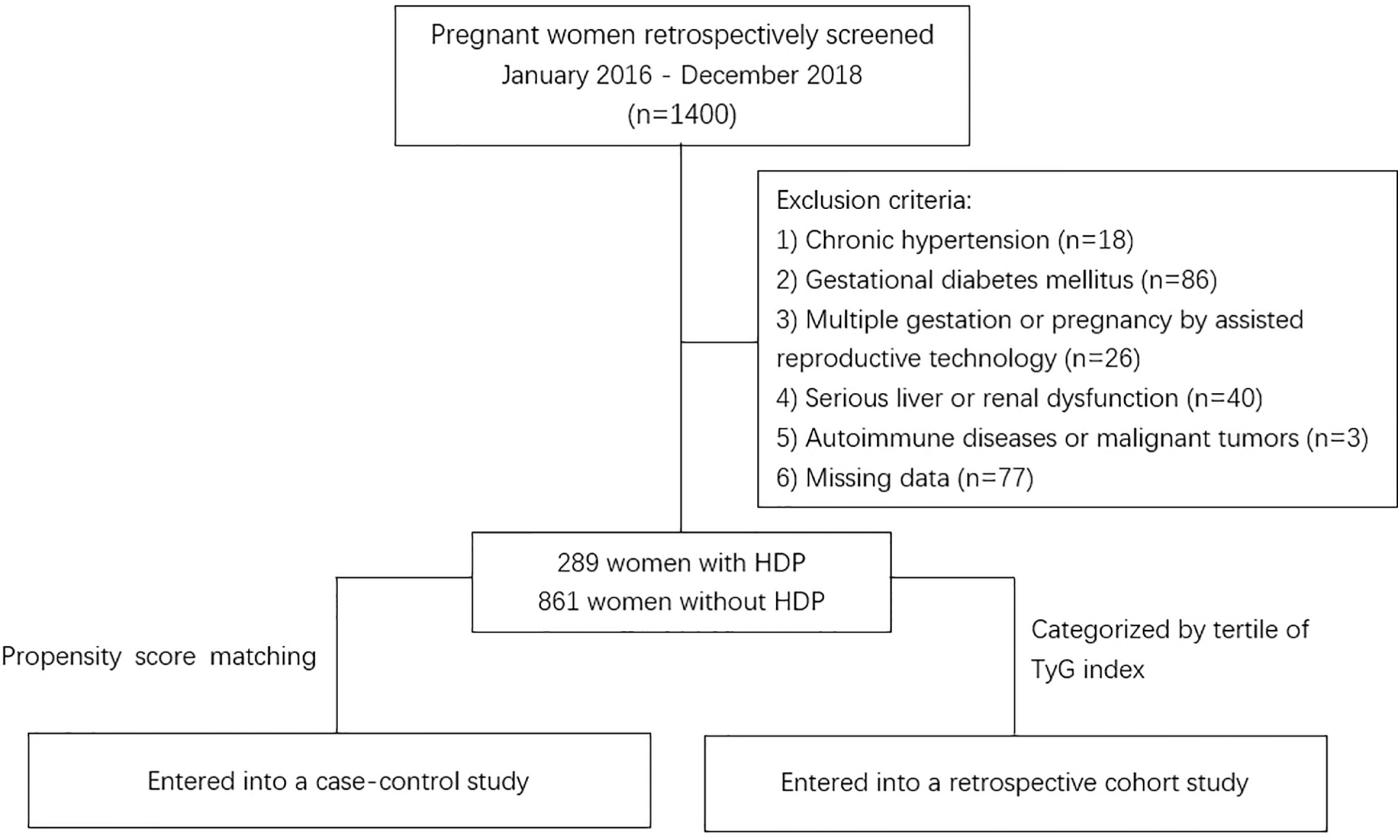
Flowchart of the study. HDP, hypertensive disorder of pregnancy; TyG, Triglyceride–glucose.

In this study, we recruited all women of Obstetrics at Shanghai Fifth People’s Hospital, Fudan University from January 2016 to December 2018, and there were 1400 pregnant women were retrospectively screened at digital medical record systems, the women had no other medical diagnosis at the beginning of pregnancy and were truly representative. Also, all the women were followed up until 12 weeks after delivery. The retrospective analysis process followed the procedure described in **Figure 1**. Women were excluded from the study for any of the following: (1) had a diagnosis of chronic hypertension before pregnancy or before 20 weeks’ gestation; (2) gestational diabetes mellitus; (3) multiple gestations or pregnancy by assisted reproductive technology; (4) serious liver dysfunction (alanine transaminase above 2.5 times upper limit) and renal dysfunction (estimated Glomerular Filtration Rate below 90 ml/min/1.73m²); (5) autoimmune diseases or malignant tumors; (6) participants with missing TyG index measurements or other data. In total, 1150 women (861 without HDP and 289 with HDP) were finally included in the analysis. The study protocol was approved by the Institutional Review Board of Shanghai Fifth People’s Hospital, Fudan University.

### Data collection

The health cards of all pregnant women were obtained from 9 to 12 weeks’ gestation and included information about age, occupation, last menstruation, method of conception, parity, obstetric history, family history of hypertension, and pre-pregnancy weight. Subsequently, at the first visit, blood pressure (BP), weight, height, blood count (Sysmex XN9000, Japan), biochemistry results (Cobas 8000, Roche, Switzerland), and fasting glucose were recorded. Fasting venous blood samples were performed after at least 8 hours of fast. The Triglyceride–glucose (TyG) index was calculated as ln[triglycerides (mg/dL) *fasting glucose (mg/dL)/2]. BP was measured with an automated sphygmomanometer (HEM-7124) in the seated position on two occasions 4h apart after resting for at least 5 minutes. The elbow of the arm used to measure BP was supported at heart level. In the presence of raised BP, routine obstetric examination was performed every 2 to 4 weeks in the outpatient clinic until 34 weeks’ gestation and thereafter every week. Each women’s body weight and height were measured in light clothing without shoes. The weighing scale, height meter, and automated sphygmomanometers were calibrated every 6 months and all measurements were taken by the same outpatient nurse. Body mass index (BMI) was calculated as weight (kg)/height (m)². After delivery, details including gestational age at delivery, mode of delivery, newborn weight, sex of the neonate and adverse pregnancy outcomes including LBW, macrosomia, preterm birth, placental abruption, postpartum hemorrhage, cesarean section, rupture of membranes, and fetal distress were recorded by medical staff.

### Definitions

HDP is a group of diseases coexisting with elevated blood pressure (systolic blood pressure (SBP) ≥ 140 mmHg and/or a diastolic blood pressure (DBP) ≥ 90 mmHg on two occasions at least 4h apart) during pregnancy (16). Preterm birth is defined as that occurring after 28 weeks and before 37 completed weeks of gestation (17). Low birth weight (LBW) is defined as that the neonates who were born with birth weights lower than 2500g (18). Macrosomia was defined as birth weight more than 4000g (18). Fetal distress is defined as a non-reassuring fetal status that the baby does not have the adequate amount of oxygen supply before labor, during the labor process or after the period of labor (19). Postpartum hemorrhage is defined as losing more than 500 milliliters of blood 24 hours after vaginal birth, and more than 1000 milliliters of blood after a cesarean birth (20).

### Outcomes

The outcome was the incidence of HDP and adverse pregnancy outcome including LBW, macrosomia, preterm birth, placental abruption, postpartum hemorrhage, cesarean section, rupture of membranes, and fetal distress.

### Statistical analysis

To avoid the confounding effects on BP between women with or without HDP, a propensity score matching (PSM) method was employed to match variables of age, pre-pregnancy BMI, family history of hypertension and parity. Matching tolerance was 0.02. We used Shapiro–Wilk test and the shape of the histogram to check the normality. Proportions (%) were used for categorical variables and mean and standard deviation (SD) or median and interquartile range (IQR) was used for continuous variables. χ2 test, the Student’s t-test and Mann-Whitney U test were used to identify the difference between groups. To determine whether TyG index was an independent risk factor, logistic regression analysis was performed with HDP classified in a binary manner (presence/absence) as the dependent variable. Receiver operating characteristic curves were performed to assess the predictability for HDP. To further validate the association of TyG index with HDP and pregnancy outcomes, a cohort study including all subjects was established in which patients were divided into three groups by tertiles of TyG index. χ2, Kruskal-Wallis and *post hoc* test were used to identify the difference in the mean between groups. Linear association between TyG index and pre-pregnancy BMI, SBP and DBP one week before delivery were assessed by simple linear regression analysis. Continuous association of TyG index with HDP incidence was determined by spline regression analysis. All analyses were performed using SPSS 26.0. A two-tailed P value < 0.05 was considered statistically significant.

## Results

### Characteristics of women with and without HDP in all subjects and subjects after PSM

In this study, HDP developed in 289 women (25.13%) among the 1150 subjects, and women with older age (*P*<0.001), higher pre-pregnancy BMI (*P*=0.002) and family history of hypertension (*P*<0.001) were more likely to develop HDP (**Table 1**). At the first visit, compared with women without HDP, patients with HDP had a much higher level of TG (1.83 ± 0.83 vs. 1.62 ± 0.63 mmol/L, *P*<0.001), FBG (4.20 ± 0.48 vs. 4.04 ± 0.40 mmol/L, *P*<0.001), TyG index (8.63 ± 0.41 vs. 8.49 ± 0.36, *P*<0.001) and WBC (8.96 ± 1.82 vs. 8.63 ± 1.98×10^9/L, *P*=0.012), whereas the difference in TC or LDL was not significant. Also, there were no significant differences in SBP, DBP, ALT, AST, and creatin at baseline between the two groups. One week before delivery, women with HDP had a much higher SBP (123 ± 14 vs. 121 ± 13 mmHg, *P*=0.022), DBP (77 ± 11 vs. 75 ± 11 mmHg, *P*=0.026), and creatinine (50.12 ± 8.83 vs. 46.39 ± 7.40 μmol/L, *P*<0.001). In addition, there were significant differences in weight gain (16.93 ± 4.72 vs. 15.79 ± 4.38 kg, *P*<0.001) during the whole pregnancy between the two groups. For pregnancy outcome, obviously, mothers with HDP tended to deliver lower-weight newborns (3220.3 ± 619.8 vs. 3376.8 ± 417.5 g, *P*<0.001), and had a higher rate of fetal distress (10.0% vs. 3.3%, *P*<0.001), cesarean section (59.5% vs. 42.9%, *P*<0.001), preterm (10.7% vs. 3.5%, *P*<0.001), postpartum hemorrhage (11.8% vs. 3.6%, *P*<0.001) and delivering low birth weight infants (10.7% vs. 2.1%, *P*<0.001) than mothers without HDP (**Table 1**).

**Table 1:** Characteristics of women with and without HDP in all subjects and subjects after PSM.

	All subjects					After PSM			
	Women without HDP	Women with HDP			*P*	Women without HDP	Women with HDP		*P*
n	861			289				230	230		
Age (years)	28.2 ± 5.6			29.7 ± 5.3			**<0.001**	28.7 ± 6.0	28.7 ± 5.0		0.933
Pre-pregnancy BMI (kg/m²)	22.0 ± 3.0			22.7 ± 3.5			**0.002**	21.5 ± 2.8	21.8 ± 3.0		0.144
Family history of hypertension									
No	772(89.7)			230(79.6)			**<0.001**	199(86.5)	202(87.8)		0.676
Yes	89(10.3)			59(20.4)				31(13.5)	28(12.2)		
Parity									
Nulliparous	352(40.9)			166(57.4)			**<0.001**	123(53.5)	113(49.1)		0.351
Parous	509(59.1)			123(42.6)				107(46.5)	117(50.9)		
At the first visit									
SBP (mmHg)	116 ± 9			116 ± 10			0.977	116 ± 8	116 ± 6		0.198
DBP (mmHg)	70 ± 8			71 ± 8			0.458	71 ± 7	71 ± 7		0.840
ALT (units/L)	14.0(10.3-18.0)			15(10.0-22.6)			0.153	14.0(11.0-18.0)	14.3(10.0-22.0)		0.508
AST (units/L)	19.0(15.0-23.0)			19.4(15.0-25.0)			0.146	19.0(15.2-24.0)	19.0(15.0-25.0)		0.585
Creatinine (μmol/L)	44.15 ± 5.74			44.47 ± 6.17			0.421	43.94 ± 5.93	44.19 ± 6.41		0.664
TC (mmol/L)	4.81 ± 0.87			4.70 ± 0.84			0.057	4.80 ± 0.87	4.69 ± 0.87		0.159
TG (mmol/L)	1.62 ± 0.63			1.83 ± 0.83			**<0.001**	1.46 ± 0.54	1.78 ± 0.72		**<0.001**
HDL (mmol/L)	1.94 ± 0.43			1.80 ± 0.38			**<0.001**	1.96 ± 0.42	1.84 ± 0.37		**0.001**
LDL (mmol/L)	2.73 ± 0.76			2.64 ± 0.72			0.072	2.62 ± 0.78	2.62 ± 0.77		0.927
FBG (mmol/L)	4.04 ± 0.40			4.20 ± 0.48			**<0.001**	4.05 ± 0.39	4.21 ± 0.49		**<0.001**
TyG index	8.49 ± 0.36			8.63 ± 0.41			**<0.001**	8.39 ± 0.35	8.62 ± 0.39		**<0.001**
WBC (*10^9/L)	8.63 ± 1.98			8.96 ± 1.82			**0.012**	8.73 ± 1.83	8.91 ± 1.83		0.282
One week before delivery									
SBP (mmHg)	121 ± 13	123 ± 14			**0.022**	120 ± 6	135 ± 12		**<0.001**
DBP (mmHg)	75 ± 11	77 ± 11			**0.026**	75 ± 6	88 ± 8		**<0.001**
ALT (units/L)	8.7(7.0-11.0)	9.0(7.0-12.1)			0.06	9.0(7.0-11.0)	9.0(7.0-12.0)		0.237
Creatinine (μmol/L)	46.39 ± 7.40	50.12 ± 8.83			**<0.001**	46.82 ± 7.00	50.43 ± 9.04		**<0.001**
TC (mmol/L)	6.12 ± 1.11	6.16 ± 1.22			0.633	6.12 ± 1.10	6.20 ± 1.25		0.494
TG (mmol/L)	3.04 ± 0.97	3.63 ± 1.26			**<0.001**	3.02 ± 0.98	3.57 ± 1.20		**<0.001**
HDL (mmol/L)	1.87 ± 0.38	1.81 ± 0.43			**0.035**	1.85 ± 0.39	1.83 ± 0.43		0.562
LDL (mmol/L)	3.64 ± 1.04	3.54 ± 1.03			0.252	3.64 ± 1.03	3.58 ± 1.02		0.542
FBG (mmol/L)	4.01 ± 0.47	4.15 ± 0.59			**0.001**	4.00 ± 0.43	4.13 ± 0.57		**0.003**
TyG index	9.13 ± 0.34	9.33 ± 0.35			**<0.001**	9.12 ± 0.34	9.31 ± 0.35		**<0.001**
WBC (*10^9/L)	9.12 ± 2.34	9.09 ± 2.05			0.845	8.92 ± 2.19	9.04 ± 2.09		0.544
Weight gain (kg)	15.79 ± 4.38	16.93 ± 4.72			**<0.001**	15.82 ± 4.59	16.92 ± 4.59		**0.011**
	All subjects					After PSM			
	Women without HDP	Women with HDP		*P*		Women without HDP		Women with HDP	*P*
n	861	289				230	230		
Pregnancy outcome									
Fetus sex (female)	422(49.9)	156(54.0)			0.229	117(50.9)	127(55.2)		0.350
Newborn weight (g)	3376.8 ± 417.5	3220.3 ± 619.8			**<0.001**	3384.8 ± 395.4	3158.4 ± 623.2		**<0.001**
LBW	18(2.1)	31(10.7)			**<0.001**	10(4.3)	25(10.9)		**0.008**
Macrosomia	47(5.5)	18(6.2)			0.624	9(3.9)	10(4.3)		0.815
Fetal distress	28(3.3)	29(10.0)			**<0.001**	9(3.9)	23(10.0)		**0.010**
Cesarean section	363(42.9)	172(59.5)			**<0.001**	93(40.4)	128(55.7)		**<0.001**
Preterm	30(3.5)	31(10.7)			**<0.001**	11(4.8)	23(10.0)		**0.032**
Rupture of membranes	102(11.8)	22(7.6)			0.058	23(10.0)	16(7.0)		0.241
Placental abruption	3(0.4)	4(1.4)			0.071	0.0(0.0)	3(1.3)		0.247
Postpartum hemorrhage	31(3.6)	34(11.8)			**<0.001**	21(9.1)	26(11.3)		0.441

Data are means ± SD, median (IQR), or n (%). *Log-transformed for t test. Boldface P values are statistically significant (P<0.05).

DBP, diastolic blood pressure; SBP, systolic blood pressure; ALT, alanine aminotransferase; AST, aspartate aminotransferase;

TC, cholesterol; TG, triglyceride; HDL, high density lipoprotein; LDL, low density lipoprotein; FBG. Fasting glucose; TyG, Triglyceride–glucose; WBC, white blood cell; LBW, low birth weight; PSM, propensity score matching.

PSM to even the variables of age, pre-pregnancy BMI, family history of hypertension and parity, match tolerance was 0.02.

To eliminate confounding effects on BP, a PSM method was employed to match variables of age, pre-pregnancy BMI, family history of hypertension, and parity. After matching, there were no significant differences in WBC at the first visit, HDL one week before delivery, and incidence of postpartum hemorrhage between women with and without HDP. Nonetheless, there remained a significantly higher TG (*P*<0.001), FBG (*P*<0,001), and TyG index (*P*<0.001) at baseline, higher SBP (*P*<0.001), DBP (*P*<0.001), and creatinine (*P*<0.001) one week before delivery and higher incidence of LBW (*P*=0.008), fetal distress (*P*=0.010), cesarean section (*P*<0.001) and preterm (*P*=0.032) in women with HDP compared with control subjects (**Table 1**).

### The early trimester TyG index was an independent risk factor and diagnostic predictive factor for HDP development, LBW and fetal distress

**Figure 2:**
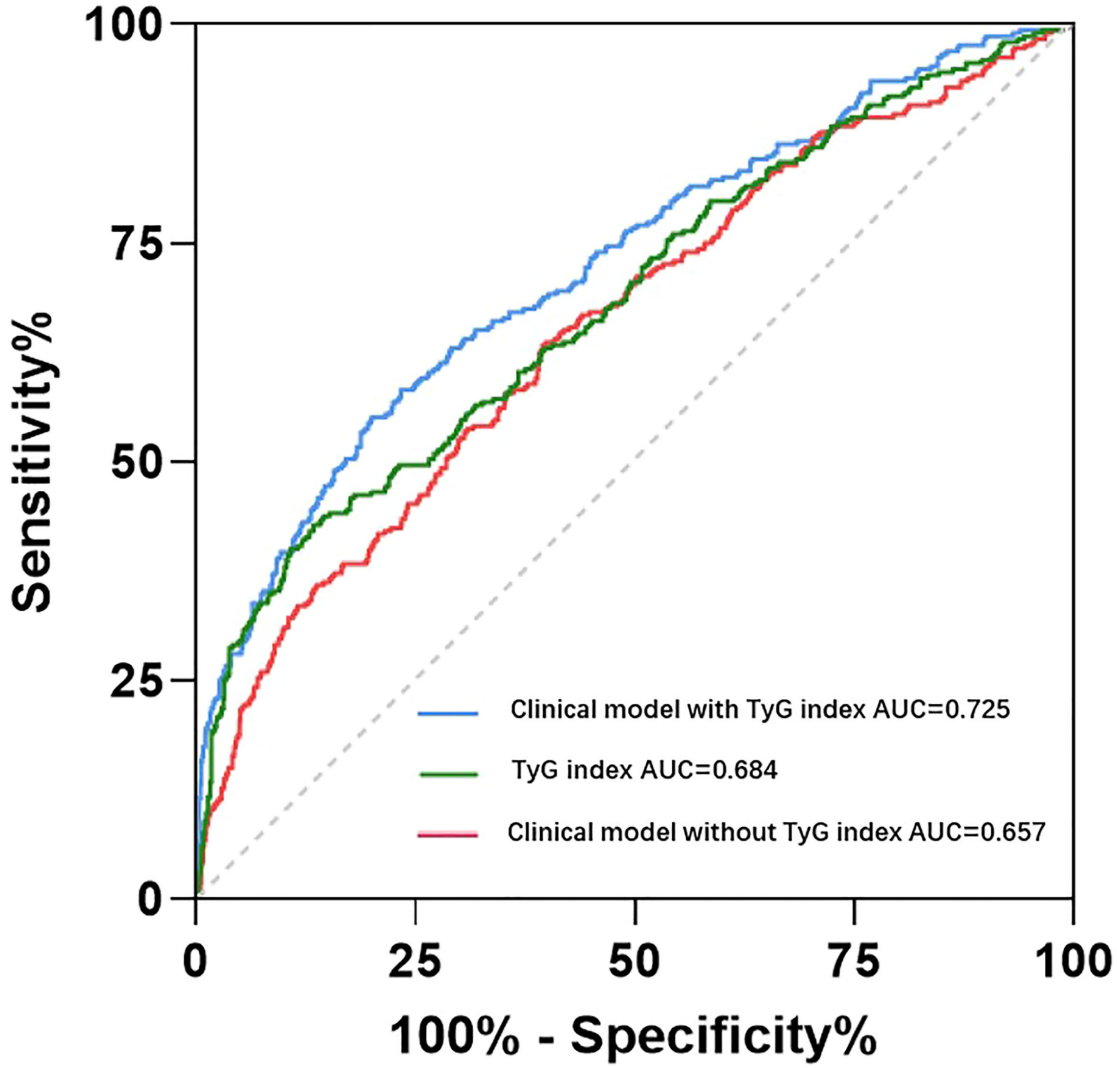
Receiver operating characteristic curve analysis of the TyG index and clinical with or without TyG index to predict incident HDP. Clinical model include age, pre-pregnancy BMI, family history of hypertension, and parity. AUC, area under curve.

In case-control study, we used multiple logistic regression to analyze the odds ratio (OR) of HDP incidence in each TyG group for all subjects. After adjusted by age, pre-pregnancy BMI, family history of hypertension, parity and weight gain, compared to the lowest tertile of the TyG index, the OR for subjects in tertile 2 and tertile 3 were 1.46 (95%CI: 1.02-2.09; *P*=0.040) and 2.44 (95%CI: 1.71-3.48; *P*<0.001) respectively (**Table 2**). The same results were showed in the logistic regression for the subjects after PSM (**Supplementary Table 1**). All of these showed that early trimester TyG index as an independent risk factor was closely correlated with the development of HDP. The ROC curves of the TyG index as a marker to predict the incidence of HDP are illustrated in **Figure 2**. Clinical model include age, pre-pregnancy BMI, family history of hypertension, and parity. The AUC of the TyG index for predicting the occurrence of HDP was 0.684 (95% CI: 0.647–0.721), but the clinical model without TyG index for predicting was 0.657 (95% CI: 0.620-0.695), adding TyG index to the model significantly increased its discriminatory capacity (AUC: 0.725; 95% CI: 0.690-0.760).

**Table 2:** Logistic regression analysis with enter selection for presence/absence HDP.

	B	OR	95%CI	*P*
TyG index (At first visit)				
Tertile 1	Reference			
Tertile 2	0.38	1.46	1.02-2.09	**0.040**
Tertile 3	0.89	2.44	1.71-3.48	**<0.001**
Age	0.06	1.06	1.03-1.10	**<0.001**
Pre-pregnancy BMI	0.06	1.06	1.01-1.11	**0.010**
Family history of hypertension	0.79	2.20	1.51-3.23	**<0.001**
Parity	-1.08	0.34	0.24-0.47	**<0.001**
Weight gain	0.05	1.05	1.02-1.10	**0.001**

TyG, Triglyceride–glucose. The bold values means statistically significant.

Besides, TyG index was also an independent risk factor for the incidence of LBW and fetal stress. After adjusted by age, pre-pregnancy BMI, family history of hypertension, parity and weight gain, compared to the lowest tertile of the TyG index, the OR for incident LBW and fetal distress in tertile 3 were 2.59 (95%CI: 1.25-5.33; *P*=0.010) and 2.92 (95%CI: 1.40-6.10; *P*=0.004) (**Supplementary Table 2**).

### Comparison of parameters during pregnancy among three groups categorized by tertiles of the early trimester TyG index in the cohort study

All subjects were divided into three groups according to tertiles of the early trimester TyG index: lowest group (<8.35), middle group (8.35-8.70), and highest group (>8.70). There was a stepwise increase in the incidence of HDP (18.9%, 23.6%, and 33.4%; *P*<0.001), age (28.1 ± 4.8 vs. 28.6 ± 5.3 vs. 29.1 ± 5.3 years; *P*=0.038), pre-pregnancy BMI (21.5 ± 2.9 vs. 22.1 ± 3.0 vs. 22.8 ± 3.3 kg/m²; *P*<0.001), DBP (70 ± 8 vs. 70 ± 8 vs. 71 ± 7 mmHg; *P*=0.003), TC (4.54 ± 0.78 vs. 4.77 ± 0.81 vs. 5.05 ± 0.93 mmol/L; *P*<0.001), TG (1.10 ± 0.19 vs. 1.55 ± 0.20 vs. 2.41 ± 0.70 mmol/L; *P*<0.001), HDL (2.00 ± 0.41 vs. 1.93 ± 0.41 vs. 1.77 ± 0.41 mmol/L; *P*<0.001), LDL (2.49 ± 0.65 vs. 2.69 ± 0.70 vs. 2.95 ± 0.83 mmol/L; *P*<0.001), FBG (3.98 ± 0.39 vs. 4.10 ± 0.40 vs. 4.18 ± 0.47 mmol/L; *P*<0.001) and WBC (8.40 ± 1.87 vs. 8.65 ± 2.01 vs. 9.12 ± 1.90 *10^9/L; *P*<0.001) at the first visit, level of SBP (116 ± 12 vs. 118 ± 9 vs. 132 ± 13 mmHg; *P*<0.001), DBP (70 ± 9 vs. 73 ± 8 vs. 85 ± 10 mmHg; *P*<0.001), TG (2.85 ± 0.94 vs. 3.26 ± 0.96 vs. 3.89 ± 1.30 mmol/L; *P*<0.001), HDL (1.93 ± 0.42 vs. 1.84 ± 0.40 vs. 1.75 ± 0.37 mmol/L; *P*<0.001) and FBG (3.98 ± 0.44 vs. 4.11 ± 0.51 vs. 4.15 ± 0.63 mmol/L; *P*=0.002) one week before delivery. Likewise, the incidence of LBW(3.1%, 3.1%, and 6.8%, *P*=0.014), fetal distress (2.8%, 5.1%, and 7.1%; *P*=0.025), cesarean section (29.4%, 46.5%, and 67.8%, *P*<0.001), preterm (4.8%, 3.6% and 7.6%, *P*=0.042) and placental abruption (0.0%, 0.5% and 1.4%, *P*=0.028) increase as TyG index increased (**Table 3**).

**Table 3:** Comparison of parameters during pregnancy among three groups categorized by tertiles of TyG index at the first visit in the cohort study.

	Tertile 1	Tertile 2	Tertile 3	*P*
TyG index range	<8.35	8.35-8.70	>8.70	
N	392	390	368	
Women with HDP	74(18.9)	92(23.6)	123(33.4)	**<0.001**
Age (years)	28.1 ± 4.8	28.6 ± 5.3	29.1 ± 5.3**†**	**0.038**
Pre-pregnancy BMI (kg/m²)	21.5 ± 2.9	22.1 ± 3.0*	22.8 ± 3.3**†#**	**<0.001**
Family history of hypertension	30(7.6)	51(13.1)	72(20.5)	**<0.001**
Parity (Nulliparous)	211(53.8)	169(43.3)	138(37.5)	**<0.001**
At the first visit				
SBP (mmHg)	116 ± 11	115 ± 10	116 ± 7	0.115
DBP (mmHg)	70 ± 8	70 ± 8	71 ± 7**†**	**0.003**
ALT (units/L)	13.2(10.0-18.8)	14.0(10.0-20.9)	13.1(9.0-20.8)	0.507
AST (units/L)	18.0(15.0-23.0)	19.0(15.0-24.0)	19.0(15.0-24.0)	0.471
Creatinine (μmol/L)	43.99 ± 5.69	44.39 ± 6.09	44.32 ± 5.76	0.754
TC (mmol/L)	4.54 ± 0.78	4.77 ± 0.81*	5.05 ± 0.93**†#**	**<0.001**
TG (mmol/L)	1.10 ± 0.19	1.55 ± 0.20*	2.41 ± 0.70**†#**	**<0.001**
HDL (mmol/L)	2.00 ± 0.41	1.93 ± 0.41	1.77 ± 0.41**†#**	**<0.001**
LDL (mmol/L)	2.49 ± 0.65	2.69 ± 0.70*	2.95 ± 0.83**†#**	**<0.001**
FBG (mmol/L)	3.98 ± 0.39	4.10 ± 0.40*	4.18 ± 0.47**†#**	**<0.001**
WBC (*10^9/L)	8.40 ± 1.87	8.65 ± 2.01	9.12 ± 1.90**†#**	**<0.001**
One week before delivery				
SBP (mmHg)	116 ± 12	118 ± 9*	132 ± 13**†#**	**<0.001**
DBP (mmHg)	70 ± 9	73 ± 8*	85 ± 10**†#**	**<0.001**
ALT (units/L)	11.0(8.0-18.0)	11.0(7.7-19.3)	11.0(7.7-19.0)	0.928
Creatinine (μmol/L)	47.42 ± 8.01	47.32 ± 7.84	47.23 ± 8.03	0.876
TC (mmol/L)	6.12 ± 1.17	6.12 ± 1.08	6.18 ± 1.16	0.840
TG (mmol/L)	2.85 ± 0.94	3.26 ± 0.96*	3.89 ± 1.30**†#**	**<0.001**
HDL (mmol/L)	1.93 ± 0.42	1.84 ± 0.40	1.75 ± 0.37**†#**	**<0.001**
LDL (mmol/L)	3.66 ± 0.99	3.59 ± 1.04	3.52 ± 1.07	0.389
FBG (mmol/L)	3.98 ± 0.44	4.11 ± 0.51*	4.15 ± 0.63**†**	**0.002**
WBC (*10^9/L)	9.19 ± 2.25	9.01 ± 2.39	9.14 ± 2.16	0.500
Weight gain	16.42 ± 4.36	15.82 ± 4.58	16.07 ± 4.50	0.166
Pregnancy outcome				
Fetus sex (female)	197(49.9)	183(47.0)	185(52.7)	0.306
Newborn weight (g)	3314.7 ± 433.2	3356.6 ± 440.3	3337.6 ± 558.4	0.327
LBW	12(3.1)	12(3.1)	25(6.8)	**0.014**
Macrosomia	15(3.8)	21(5.4)	29(7.9)	0.052
Fetal distress	11(2.8)	20(5.1)	26(7.1)	**0.025**
Cesarean section	116(29.4)	181(46.5)	238(67.8)	**<0.001**
Preterm	19(4.8)	14(3.6)	28(7.6)	**0.042**
Rupture of membranes	47(12.0)	40(10.3)	37(10.1)	0.635
Placental abruption	0(0.0)	2(0.5)	5(1.4)	**0.028**
Postpartum hemorrhage	23(5.9)	15(3.8)	27(7.3)	0.112

Data are means ± SD, median (IQR), or n (%). *Log-transformed for t test. Boldface P values are statistically significant (P<0.05).

DBP, diastolic blood pressure; SBP, systolic blood pressure; ALT, alanine aminotransferase; AST, aspartate aminotransferase;

TC, cholesterol; TG, triglyceride; HDL, high density lipoprotein; LDL, low density lipoprotein; FBG. Fasting glucose; TyG, Triglyceride–glucose; WBC, white blood cell; LBW, low birth weight. *Middle group vs. lowest group, P<0.05. †Highest

group vs. lowest group, P<0.05. #Highest group vs. middle group, P<0.05.z

### The early trimester TyG index was closely associated with pre-pregnancy BMI, SBP and DBP one week before delivery

**Figure 3:**
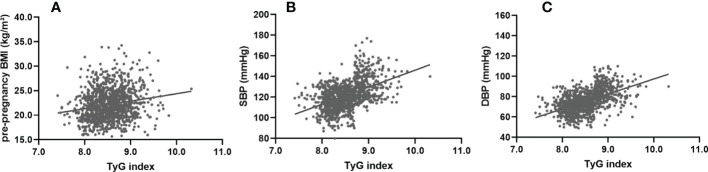
Simple linear regression analysis between the TyG index at first visit and pre-pregnancy BMI, SBP and DBP one week before delivery. The TyG index showed a significant and moderate linear association with pre-pregnancy BMI (β=0.31; F=65.21; adjusted R²=0.09; P<0.001) **(A)**, SBP (β=0.25; F=41.48; adjusted R²=0.06; P<0.001) **(B)**, and DBP (β=0.38; F=106.11; adjusted R²=0.14; P<0.001) **(C)**. SBP, systolic blood pressure; DBP, diastolic blood pressure.

To investigate the correlation between the early trimester TyG index and pre-pregnancy BMI, SBP or DBP one week before delivery, correlation analysis was performed. Simple linear regression analyses were performed to determine the association of the early trimester TyG index with pre-pregnancy BMI, SBP and DBP one week before delivery. There was a significant and moderate linear association for TyG index with pre-pregnancy BMI [β=0.18; t=6.35; *P*<0.001] (**Figure 3A**), SBP [β=0.47; t=17.78; *P*<0.001] (**Figure 3B**), and DBP [β=0.51; t=20.00; *P*<0.001] (**Figure 3C**).

### The early trimester TyG index was closely associated with the incidence of HDP

**Figure 4:**
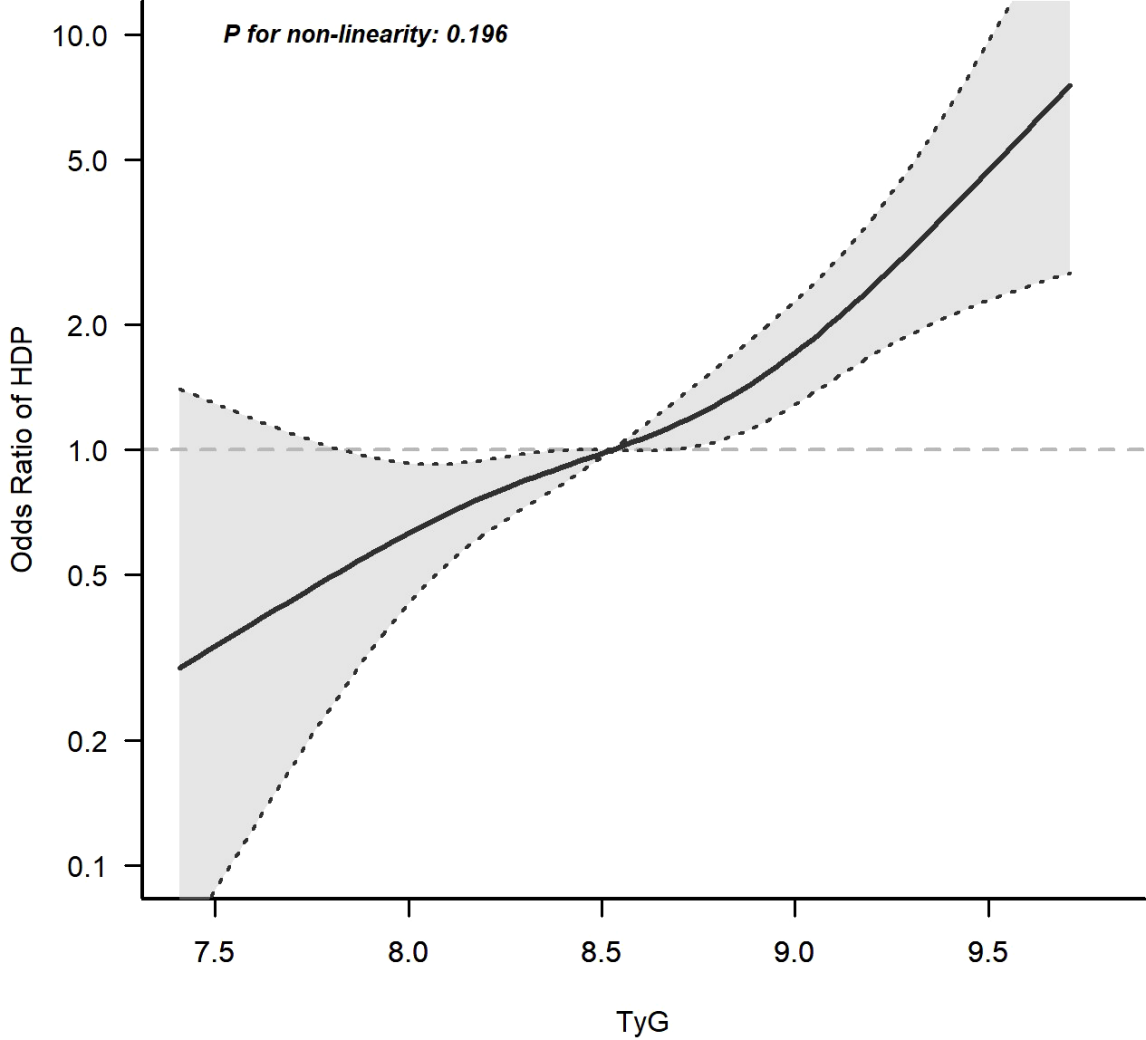
Continuous association of the TyG index at the first visit with the incidence of HDP. Adjusted for age, pre-pregnancy BMI, family history of hypertension and parity. The risk of developing HDP increased when the TyG index was greater than 8.5.

After adjusting for age, pre-pregnancy BMI, family history of hypertension and parity, a spline model showed a significant relationship between continuous early trimester TyG index and incidence of HDP. We found an increasing trend of incidence of HDP with a higher TyG index despite the lack of a linear relationship between the TyG index and the incidence of HDP. The risk of developing HDP increased when the TyG index was greater than 8.5 (**Figure 4**).

## Discussion

This retrospective study is the one to confirm the significant positive association and a dose-response relation between the early trimester TyG index and the incidence of HDP in a large sample size. Furthermore, the results provide evidence that the early trimester TyG index is independently associated with the incidence of LBW and fetal distress. This study elucidated the substantial role of TyG index in predicting the development of HDP and adverse pregnancy outcome.

Pregnancy is characterized by a number of metabolic adaptations. Insulin resistance (IR) is a normal characteristic of pregnancy and increases physiologically as the pregnancy progresses to support the normal fetal development and growth (21). Some studies demonstrated the mechanism of IR in pregnancy, Daniela et al. showed that some steroid hormones which are elevated in pregnancy, such as progesterone and corticosteroids, contribute to impaired insulin sensitivity and glucose tolerance (22), Marilyn et al. showed that some cytokines and hormones secreted by the placenta including leptin and TNFα could be implicated in IR during pregnancy (21). Some markers correlate with IR, such as triglycerides, free fatty acids, small dense LDL particles and PAI-1, increase as normal pregnancy and associated IR progress (23, 24). Moreover, a growing number of studies have described the central role of IR in development of HDP. In a study using the euglycemic clamp technique, compared with controls, women with gestational hypertension exhibited approximately 40% lower steady-state insulin sensitivity index and approximately 33% higher mean plasma TG (25). IR may increase gestational blood pressure through activation of the sympathetic nervous system (8, 26), sodium reabsorption by the distal nephron segments (6), enhanced vascular resistance and endothelial dysfunction (26, 27). Furthermore, the recognition that features of insulin resistance persists many years after pregnancy among women which raises the risk for future cardiovascular disease (28–30). These observations suggest that interventions to reduce insulin resistance may reduce the risk of both hypertension in pregnancy and later life cardiovascular complications.

The TyG index, the product of fasting glucose and triglycerides, which has high sensitivity (96.5%) and specificity (85.0%; AUC: 0.858) for diagnosis of IR (31), is a simple, reliable and early marker of IR, and can be widely used in clinical practice especially in primary hospital because all clinical laboratories can measure triglycerides and glucose and quantification of insulin levels is not required. Besides, Ana Carolina et al. showed that the TyG index had a slightly better performance compared with the HOMA-IR in identifying patients with IR (13). Although there were few studies on the relationship between the TyG index and HDP, in the study of the TyG index and hypertension, Yi Wang et al. showed that compared with those with the lowest category of T yG index, subjects with the highest category of TyG index were associated with higher odds of hypertension (32), the reason for its lower risk ratio than in our study may be that the degree of IR is more pronounced during pregnancy. In addition, in a longitudinal population-based study, Zheng et al. revealed that the TyG index can predict the incidence of hypertension among the Chinese population (15), after 9 years follow up, compared with the lowest TyG group, the hazard ratios for subjects in quartile 2, quartile 3 and quartile 4 increased, and were statistically significant. And numerous studies have also found that the TyG index was closely associated with hypertension (33–35). A retrospective cohort study showed that High maternal triglyceride level had higher risks of HDP in all maternal FPG strata, and both the early-pregnancy FPG and mTG levels should be screened among overall population including the low-risk population to reduce the incidence of pregnancy complications (36). The TyG index, which is the product of FBG and TG, may have a more obvious association with HDP.

In our investigation, to avoid confounding, a PSM method was applied and revealed that women with HDP had a much higher level of the early trimester TyG index. We also confirmed that the TyG index was independently associated with the incidence of HDP and was a diagnostic predictive factor for HDP development in case-control study. Further analysis of the relationship between the early trimester TyG index and HDP was performed in the cohort study. We found the incidence of HDP increased progressively with the increase of the early trimester TyG index and the risk of developing HDP increased when the TyG index was greater than 8.5. All of these indicated a close relation between the early trimester TyG index and HDP development.

Another finding in our study was that the TyG index was also associated with adverse pregnancy outcome. In the case-control study, we found that a higher TyG index was an independent risk factor for LBW and fetal distress. In the cohort study, women with the highest tertile of the TyG index had the highest risk for cesarean section, preterm and placental abruption. Maternal hyperglycemia and obesity predispose offspring to metabolic dysfunction (37), although the underlying mechanism is elusive, and the fetal hyperglycemia and IR disrupt normal surfactant synthesis and function, which may lead to adverse outcome in neonates (38). In addition, the women with higher blood pressure have decreased uteroplacental blood flow, which obstructs fetal growth and exacerbates fetal hypoxia and ischemia (39), thereby increases the risk of fetal distress and the need for timely termination of pregnancy.

There were some limitations to our study. First, all subjects were derived from one center, which may have led to biased results. Second, we did not measure dietary sodium intake and physical activity which may affect the blood pressure or lipids. Third, this is a retrospective study, due to the lack of information, fasting insulin was not obtained. Also, the TyG index is associated with gestational diabetes and the incidence of post-partum diabetes in women, and we are collecting data on women with gestational diabetes, we would like to explore the relationship between gestational diabetes and HDP once we have data. Despite these limitations, this study first demonstrated the association between the TyG index and HDP development.

## Conclusions

In conclusion, our study suggested that the early trimester TyG index was closely associated with HDP development and adverse pregnancy outcomes. Besides, the TyG index could be a novel and clinically effective indicator for identifying the risk of HDP especially in primary hospital.

## Data availability statement

The raw data supporting the conclusions of this article will be made available by the authors, without undue reservation.

## Ethics statement

The studies involving human participants were reviewed and approved by Medical Ethics Committee of Shanghai Fifth People’s Hospital Affiliated to Fudan University. Written informed consent for participation was not required for this study in accordance with the national legislation and the institutional requirements.

## Author contributions

YP and GZ designed the study. YP, SZ, ZW, CY and XW collected the data. YP, RD and HG analyzed the data. YP wrote the main manuscript text. GZ and YX contributed to the refinement of the manuscript. All authors contributed to the article and approved the submitted version.

## Supplementary material

Supplementary Table 1 Logistic regression analysis for presence/absence HDP in subjects after PSM

Supplementary Table 2 Logistic regression analysis to determine the risk factor for LBW/fetal distress

## References

[1] Magee LA Smith GN Bloch C Côté AM Jain V Nerenberg K. Guideline no. 426: Hypertensive disorders of pregnancy: Diagnosis, prediction, prevention, and management. J obstet gynaecol Canada JOGC = J d'obstetrique gynecol du Canada JOGC. (2022) 44(5):547–71.e1. doi: 10.1016/j.jogc.2022.03.002 35577426

[2] Cífková R. Cardiovascular sequels of hypertension in pregnancy. J Am Heart Assoc (2018) 7(10):e009300. doi: 10.1161/JAHA.118.009300 29755035 PMC6015310

[3] Ye C Ruan Y Zou L Li G Li C Chen Y. The 2011 survey on hypertensive disorders of pregnancy (HDP) in China: prevalence, risk factors, complications, pregnancy and perinatal outcomes. PloS One (2014) 9(6):e100180. doi: 10.1371/journal.pone.0100180 24937406 PMC4061123

[4] Li F Qin J Zhang S Chen L. Prevalence of hypertensive disorders in pregnancy in China: A systematic review and meta-analysis. Pregnancy hypertens (2021) 24:13–21. doi: 10.1016/j.preghy.2021.02.001 33626437

[5] Rowe JW Young JB Minaker KL Stevens AL Pallotta J Landsberg L. Effect of insulin and glucose infusions on sympathetic nervous system activity in normal man. Diabetes (1981) 30(3):219–25. doi: 10.2337/diab.30.3.219 7009270

[6] Doria A Fioretto P Avogaro A Carraro A Morocutti A Trevisan R. Insulin resistance is associated with high sodium-lithium countertransport in essential hypertension. Am J Physiol (1991) 261(6 Pt 1):E684–91. doi: 10.1152/ajpendo.1991.261.6.E684 1767828

[7] Gibbons GH Dzau VJ. The emerging concept of vascular remodeling. New Engl J Med (1994) 330(20):1431–8. doi: 10.1056/NEJM199405193302008 8159199

[8] Reaven GM Lithell H Landsberg L. Hypertension and associated metabolic abnormalities–the role of insulin resistance and the sympathoadrenal system. New Engl J Med (1996) 334(6):374–81. doi: 10.1056/NEJM199602083340607 8538710

[9] Seely EW Solomon CG. Insulin resistance and its potential role in pregnancy-induced hypertension. J Clin Endocrinol Metab (2003) 88(6):2393–8. doi: 10.1210/jc.2003-030241 12788833

[10] Li X Li G Cheng T Liu J Song G Ma H. Association between triglyceride-glucose index and risk of incident diabetes: a secondary analysis based on a Chinese cohort study : TyG index and incident diabetes. Lipids Health Dis (2020) 19(1):236. doi: 10.1186/s12944-020-01403-7 33161902 PMC7649000

[11] Xuan X Hamaguchi M Cao Q Okamura T Hashimoto Y Obora A. U-Shaped association between the triglyceride-glucose index and the risk of incident diabetes in people with normal glycemic level: A population-base longitudinal cohort study. Clin Nutr (Edinburgh Scotland) (2021) 40(4):1555–61. doi: 10.1016/j.clnu.2021.02.037 33743291

[12] Pranata R Huang I Irvan Lim MA Vania R. The association between triglyceride-glucose index and the incidence of type 2 diabetes mellitus-a systematic review and dose-response meta-analysis of cohort studies. Endocrine (2021) 74(2):254–62. doi: 10.1007/s12020-021-02780-4 34086260

[13] Vasques AC Novaes FS de Oliveira Mda S Souza JR Yamanaka A Pareja JC. TyG index performs better than HOMA in a Brazilian population: a hyperglycemic clamp validated study. Diabetes Res Clin Pract (2011) 93(3):e98–e100. doi: 10.1016/j.diabres.2011.05.030 21665314

[14] Wang S Shi J Peng Y Fang Q Mu Q Gu W. Stronger association of triglyceride glucose index than the HOMA-IR with arterial stiffness in patients with type 2 diabetes: a real-world single-centre study. Cardiovasc Diabetol (2021) 20(1):82. doi: 10.1186/s12933-021-01274-x 33888131 PMC8063289

[15] Zheng R Mao Y. Triglyceride and glucose (TyG) index as a predictor of incident hypertension: a 9-year longitudinal population-based study. Lipids Health Dis (2017) 16(1):175. doi: 10.1186/s12944-017-0562-y 28903774 PMC5598027

[16] Bethesda Maryland. Report of the national high blood pressure education program working group on high blood pressure in pregnancy. Am J obstet gynecol (2000) 183(1):S1–s22. doi: 10.1067/mob.2000.107928 10920346

[17] Goldenberg RL Culhane JF Iams JD Romero R. Epidemiology and causes of preterm birth. Lancet (London England) (2008) 371(9606):75–84. doi: 10.1016/S0140-6736(08)60074-4 18177778 PMC7134569

[18] Lewandowska M. Maternal obesity and risk of low birth weight, fetal growth restriction, and macrosomia: Multiple analyses. Nutrients (2021) 13(4):1213. doi: 10.3390/nu13041213 PMC8067544 33916963

[19] van Geijn HP Copray FJ Donkers DK Bos MH. Diagnosis and management of intrapartum fetal distress. Eur J obstet gynecol Reprod Biol (1991) 42(Suppl):S63–72. 1809612

[20] Sentilhes L Merlot B Madar H Sztark F Brun S Deneux-Tharaux C. Postpartum haemorrhage: prevention and treatment. Expert review of hematology (2016) 9(11):1043–61. doi: 10.1080/17474086.2016.1245135 27701915

[21] Lacroix M Kina E Hivert MF. Maternal/fetal determinants of insulin resistance in women during pregnancy and in offspring over life. Curr Diabetes Rep (2013) 13(2):238–44. doi: 10.1007/s11892-012-0360-x 23307191

[22] Vejrazkova D Vcelak J Vankova M Lukasova P Bradnova O Halkova T. Steroids and insulin resistance in pregnancy. J Steroid Biochem Mol Biol (2014) 139:122–9. doi: 10.1016/j.jsbmb.2012.11.007 23202146

[23] Sattar N Greer IA Rumley A Stewart G Shepherd J Packard CJ. A longitudinal study of the relationships between haemostatic, lipid, and oestradiol changes during normal human pregnancy. Thromb haemost (1999) 81(1):71–5. 9974378

[24] Belo L Caslake M Gaffney D Santos-Silva A Pereira-Leite L Quintanilha A. Changes in LDL size and HDL concentration in normal and preeclamptic pregnancies. Atherosclerosis (2002) 162(2):425–32. doi: 10.1016/S0021-9150(01)00734-1 11996963

[25] Caruso A Ferrazzani S De Carolis S Lucchese A Lanzone A De Santis L. Gestational hypertension but not pre-eclampsia is associated with insulin resistance syndrome characteristics. Hum Reprod (Oxford England) (1999) 14(1):219–23. doi: 10.1093/humrep/14.1.219 10374124

[26] Fonseca VA. Insulin resistance, diabetes, hypertension, and renin-angiotensin system inhibition: reducing risk for cardiovascular disease. J Clin hypertens (Greenwich Conn) (2006) 8(10):713–20; quiz 21-2. doi: 10.1111/j.1524-6175.2006.05583.x PMC8109563 17028485

[27] Smith MM Minson CT. Obesity and adipokines: effects on sympathetic overactivity. J Physiol (2012) 590(8):1787–801. doi: 10.1113/jphysiol.2011.221036 PMC3573303 22351630

[28] Jarvie JL Metz TD Davis MB Ehrig JC Kao DP. Short-term risk of cardiovascular readmission following a hypertensive disorder of pregnancy. Heart (British Cardiac Society) (2018) 104(14):1187–94. doi: 10.1136/heartjnl-2017-312299 PMC7061962 29326108

[29] Arnott C Nelson M Alfaro Ramirez M Hyett J Gale M Henry A. Maternal cardiovascular risk after hypertensive disorder of pregnancy. Heart (British Cardiac Society) (2020) 106(24):1927–33. doi: 10.1136/heartjnl-2020-316541 32404402

[30] Garovic VD White WM Vaughan L Saiki M Parashuram S Garcia-Valencia O. Incidence and long-term outcomes of hypertensive disorders of pregnancy. J Am Coll Cardiol (2020) 75(18):2323–34. doi: 10.1016/j.jacc.2020.03.028 PMC7213062 32381164

[31] Guerrero-Romero F Simental-Mendía LE González-Ortiz M Martínez-Abundis E Ramos-Zavala MG Hernández-González SO. The product of triglycerides and glucose, a simple measure of insulin sensitivity. comparison with the euglycemic-hyperinsulinemic clamp. J Clin Endocrinol Metab (2010) 95(7):3347–51. doi: 10.1210/jc.2010-0288 20484475

[32] Wang Y Yang W Jiang X. Association between triglyceride-glucose index and hypertension: A meta-analysis. Front Cardiovasc Med (2021) 8:644035. doi: 10.3389/fcvm.2021.644035 34136539 PMC8200397

[33] Jian S Su-Mei N Xue C Jie Z Xue-Sen W. Association and interaction between triglyceride-glucose index and obesity on risk of hypertension in middle-aged and elderly adults. Clin Exp hypertens (New York NY 1993) (2017) 39(8):732–9. doi: 10.1080/10641963.2017.1324477 28737433

[34] Wang K He G Zhang Y Yin J Yan Y Zhang Y. Association of triglyceride-glucose index and its interaction with obesity on hypertension risk in Chinese: a population-based study. J Hum hypertens (2021) 35(3):232–9. doi: 10.1038/s41371-020-0326-4 32203074

[35] Wang RN Zhang DS Bai Z Yin C Zhang R Yang JL. [Prospective cohort study of relationship of triglyceride, fasting blood-glucose and triglyceride glucose product index with risk of hypertension]. Zhonghua liu xing bing xue za zhi = Zhonghua liuxingbingxue zazhi (2021) 42(3):482–7. doi: 10.3760/cma.j.cn112338-20200401-00491 34814417

[36] Wu D Zhang J Xiong Y Wang H Lu D Guo M. Effect of maternal glucose and triglyceride levels during early pregnancy on pregnancy outcomes: A retrospective cohort study. Nutrients (2022) 14(16):3295. doi: 10.3390/nu14163295 PMC9414844 36014801

[37] Oben JA Mouralidarane A Samuelsson AM Matthews PJ Morgan ML McKee C. Maternal obesity during pregnancy and lactation programs the development of offspring non-alcoholic fatty liver disease in mice. J Hepatol (2010) 52(6):913–20. doi: 10.1016/j.jhep.2009.12.042 20413174

[38] Yildiz Atar H Baatz JE Ryan RM. Molecular mechanisms of maternal diabetes effects on fetal and neonatal surfactant. Children (Basel Switzerland) (2021) 8(4):281. doi: 10.3390/children8040281 PMC8067463 33917547

[39] Berhe AK Ilesanmi AO Aimakhu CO Mulugeta A. Effect of pregnancy induced hypertension on adverse perinatal outcomes in tigray regional state, Ethiopia: a prospective cohort study. BMC pregnancy childbirth (2019) 20(1):7. doi: 10.1186/s12884-019-2708-6 31892353 PMC6938605

