# Supplementary material for: Is there any association between early trimester Triglyceride–glucose index and incidence of hypertensive disorder of pregnancy and adverse pregnancy outcomes?
Source: Front Endocrinol (Lausanne). 2023 Mar 6;14:1093991. doi: 10.3389/fendo.2023.1093991 (PMC10025371; doi:10.3389/fendo.2023.1093991)
Supplement: Supplementary Table 1 — Logistic regression analysis for presence/absence HDP in subjects after PSM [file Table_1.pdf]

Supplementary Table 1 Logistic regression analysis for presence/absence HDP in subjects after PSM

	B	OR	95%CI	*P*
TyG index (At first visit)				
Tertile 1	Reference			
Tertile 2	0.77	2.17	1.36-3.45	**0.001**
Tertile 3	1.39	4.02	2.46-6.57	**<0.001**
Age	-0.01	0.67	0.95-1.03	0.674
Pre-pregnancy BMI	0.03	1.03	0.95-1.10	0.500
Family history of hypertension	-0.11	0.90	0.50-1.60	0.708
Parity	0.05	1.05	0.64-1.73	0.839
Weight gain	0.07	1.07	1.02-1.12	**0.003**

TyG, Triglyceride–glucose.

Supplementary Table 2 Logistic regression analysis to determine the risk factor for LBW/fetal distress

	B	OR	95%CI	*P*
Logistic regression analysis for LBW				
TyG index (At first visit)				
Tertile 1	Reference			
Tertile 2	0.07	1.07	0.47-2.43	0.872
Tertile 3	0.95	2.59	1.26-5.33	**0.010**
Age	0.01	1.01	0.94-1.07	0.855
Pre-pregnancy BMI	-0.08	0.93	0.84-1.03	0.141
Family history of hypertension	0.62	1.86	0.90-3.86	0.095
Parity	-0.16	0.85	0.44-1.66	0.641
Weight gain	-0.01	0.99	0.93-1.06	0.854
Logistic regression analysis for fetal distress				
TyG index (At first visit)				
Tertile 1	Reference			
Tertile 2	0.70	2.01	0.94-4.28	0.071
Tertile 3	1.07	2.92	1.40-6.10	**0.004**
Age	-0.03	0.97	0.91-1.03	0.289
Pre-pregnancy BMI	-0.03	0.97	0.89-1.07	0.546
Family history of hypertension	0.61	1.83	0.91-3.68	0.088
Parity	0.02	1.02	0.55-1.88	0.951
Weight gain	0.09	1.09	1.03-1.16	**0.003**

TyG, Triglyceride–glucose; LBW, low birth weight.
